# Development of allergic airway inflammation in early life – interaction of early viral infections and allergic sensitization 

**DOI:** 10.5414/ALX01635E

**Published:** 2018-09-01

**Authors:** E. Hamelmann

**Affiliations:** Klinik für Kinder- und Jugendmedizin, Ruhr-Universität Bochum

**Keywords:** allergic airway inflammation, remodeling, persistent asthma, virus-triggered exacerbation

## Abstract

Airway inflammation is a key feature of upper and lower respiratory allergic diseases, such as allergic rhinitis and asthma. Characteristically, histological alterations such as goblet cell hyperplasia, mucus hypersecretion, loss of epithelial barrier function, airway infiltration and structural changes such as basal membrane thickening and airway smooth muscle hyperplasia. These inflammatory signs are often obvious already early in life and may be accompanied by structural changes (remodeling) occurring in early lifetime. This review focusses on the main mechanisms underlying the development of airway inflammation and remodeling and discusses the question which factors contribute to the persistence of airway inflammation in chronic allergic airway disease.

**German version published in Allergologie, Vol. 36, No. 11/2013, p. 486-491**

The classic and still valid model of the development of allergic airway inflammation assumes an early sensitization, which, after penetration by the allergen through the airway epithelium and the absorption of professional antigen-presenting cells (APCs), leads to the activation of type 2 T-helper cells (Th2). This initial step, together with the release of Th2 cytokines (interleukin (IL) 4, 5, 6, 13), leads, on the one hand, to the activation of eosinophils and mast cells from the bone marrow, and, on the other, to their infiltration into the pulmonary tissue and to the activation of IgE-producing plasma cells from B-cells. After re-exposure to the same allergen, the classic IgE-mediated immediate-type reaction occurs, which is characterized by bronchial obstruction, hypersecretion, and hyperpermeability/edema [12]. It was commonly assumed that this allergic inflammation and its alternation with episodes of recovery would lead to structural changes (scarring), which are termed “remodeling”. Today, this strictly sequential idea of early inflammation followed by structural remodeling has been questioned because current publications suggest very early structural changes in the pulmonary tissue in patients who later developed asthma; these structural changes were also observed without the classic allergic inflammatory response. 

This paper focuses on the mechanisms of (i) the development of airway inflammation, (ii) structural remodeling, and (iii) the chronification/persistence of airway inflammation. 

## What are the underlying mechanisms of airway inflammation? 

**Figure 1.**
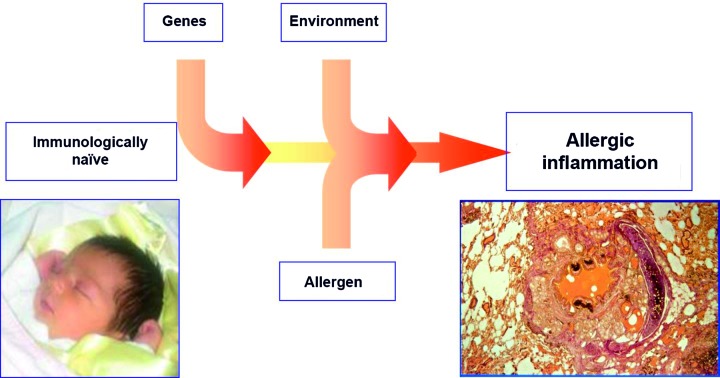
Development of allergic airway inflammation.

In general, three components contribute to the development of allergic airway inflammation (Figure 1):

- genetic predisposition,
- allergic sensitization,
- environmental factors (aggravating or protective factors).

### Genetics 

Currently, seven genome-wide studies (GWAS) on the association of genes/gene polymorphisms with the development or presence of asthma, in which also asthma during childhood and adolescence was investigated, are available [4]. These seven studies could identify a total of eleven “asthma genes”. In all of these studies, the highest asthma risk was associated with very rare variants that were only present in a very small subgroup of the study population. Only very few variants could be reproduced in all or most of the studies; one example is the gene encoding for interleukin 33, whose exact role in asthma development has not yet been fully explained. One of these studies investigated the time of asthma development. The researchers found that the onset of the disease was accelerated by 1 year on average (2.5 vs. 3.4 years of age) if two certain variants were present simultaneously [5]. In conclusion, these studies show that asthma is a genetically highly complex disease in which hundreds of genes or gene variants are involved; thus, asthma can be classified into various subgroups according to different genotypes and, in the end, asthma might not even be one single disease entity! 

The impact of genetic factors on the development of allergic airway inflammation becomes even more complex when gene expression is considered. Experimental data observed in a murine model of bronchial asthma showed that in presensitized mice, acute or chronic allergic airway exposure led to the up- or down-regulation of very different genes [15]. In this context, several genes could be identified that were found exclusively after a specific type of exposure, while other genes were turned on or off at all three time points. This is also evidence for the fact that asthma is highly complex: Hundreds of “asthma genes” are involved, and these can be modulated by environmental factors and by exposure to infectious or allergic molecules. 

### Inflammation 

**Figure 2.**
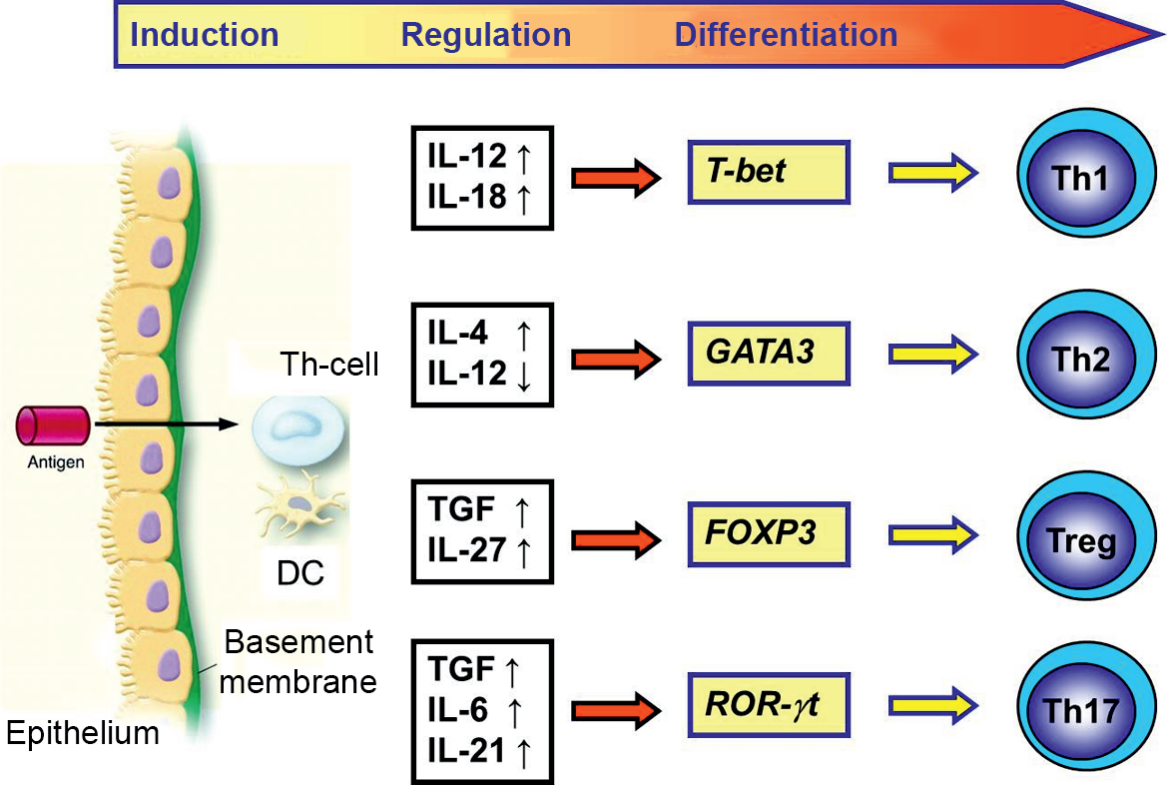
Allergic immune reaction. After absorption by the epithelium, dendritic cells (DC) present the allergen to T-helper cells, which, mediated by transcription factors, differentiate into T-effector cells (Th1, Th2, Th17) or regulatory T cells (Treg).

In the development of allergic airway inflammation, several types of cells play an important role (Figure 2). 

The first contact is mediated by the airway epithelium (AE), which has much more than a mere barrier function - it is actively involved in the process. By means of protease-activated receptors and Toll-like receptors, the AE is able to recognize allergens and allergen fragments directly. As a reaction to this, the AE itself produces proinflammatory mediators (IL-1, GM-CSF, IL-25, IL-33). These mediators activate dendritic cells (DCs), the professional APCs directly below the epithelium, and guide them into a proallergic direction. The type of immune reaction after presentation by the DCs is mainly determined by the type of Th-cells that are predominantly activated (Figure 2). 

The major role played by regulatory T-cells (Treg) in the suppression of allergic inflammation has been confirmed in experimental murine models and in studies on patients with early sensitization or manifestation of bronchial asthma [7, 20]. Thus, an important mechanism in the early development of allergic airway inflammation is the imbalance between activated proallergic Th2 cells and not enough or functionally-inactive Treg. 

## What are the underlying mechanisms of the early development of airway remodeling? 

Traditionally, the structural changes in the airways were interpreted to be a kind of scarring due to the alternation between inflammation and recovery. In the meantime, there has been increasing evidence for the fact that the constantly-present airway inflammation, which is, e.g., regularly triggered by infectious exacerbations, very soon leads to changes of the airway structure (remodeling). According to this theory, airway inflammation and remodeling occur simultaneously and together participate in the pathogenesis of asthma development, already at early stages. This hypothesis is supported by various clinical trials in which pulmonary biopsies were taken very early in life (in some cases as early as the 1st year of life) in order to correlate the histological changes with the presence of current or later signs suggestive of bronchial asthma. In detail, these studies show:

- Signs of airway remodeling, like a thickening of the basement membrane or hyperplasia of the airway smooth muscle, are already present in infants who are later diagnosed as having bronchial asthma [14, 19].
- The occurrence of remodeling is also predictive of a later development of clinical bronchial asthma [17].
- In school-aged children, pronounced remodeling is particularly observed in cases of therapy-resistant, severe, or poorly-controlled asthma [2].
- Remodeling is observed independently of eosinophilic airway inflammation or increased Th2 activity [1].

In conclusion, airway remodeling plays a crucial role in allergic airway inflammation, even if it occurs as early as preschool age. Furthermore, airway remodeling might also be partially responsible for the development of severe forms of asthma. 

## Which mechanisms lead to persistence/chronification of airway inflammation? 

One important question is which mechanisms lead to the chronification of airway inflammation as the pathological basis of persistent asthma. We know that most small children who, particularly in winter, have a tendency to develop episodes of obstructive bronchitis after viral infections, no longer show these symptoms after they have reached school age. On the other hand, predominantly children with early atopy (food allergy, sensitization to respiratory allergens) develop lasting symptoms of persistent bronchial asthma [21]. However, early allergic sensitization alone is not a sufficient parameter to predict persistent asthma with a high probability. Other important factors are environmental influences that lead to the exacerbation of allergic airway inflammation. 

Evidence from large clinical studies and birth cohorts demonstrating the high impact of early viral infections, particularly those caused by human rhinoviruses (HRV) and respiratory synsytial viruses (RSV), has become so strong that a dual mechanism of asthma development – allergic plus virus-triggered persistent airway inflammation – can be assumed. 

This is mainly based on the following observations:

- Lower airway infections in fall and winter are the main reason for obstructive bronchitis [11].
- Large-scale birth cohort studies have demonstrated that early viral infections of the lower airways are an important risk factor for the later development of bronchial asthma [8].
- Furthermore, prospective studies, in particular large-scale birth cohorts, have shown that these lower-airway viral infections lead to persistent asthma predominantly in those children with a history of allergic sensitization/atopy [3].
- In addition, recent prospective studies have shown that a loss of symptom control due to viral infections mainly occurs in allergic children and adolescents with asthma [16] and that the risk of asthma exacerbation is directly correlated with the level of atopy [10].
- Finally, it has been demonstrated that in allergic patients, molecules of the allergic cascade (IL-4, IL-13, IgE receptor) are directly triggered and up-regulated by viral infections of the lower airways [9].

In conclusion, these results clearly illustrate that: allergic sensitization and viral infections are two independent risk factors for the development of bronchial asthma; their interaction significantly increases the risk; and from a pathophysiological point of view, they are both involved in the development of chronic airway inflammation and airway remodeling. 

**Figure 3.**
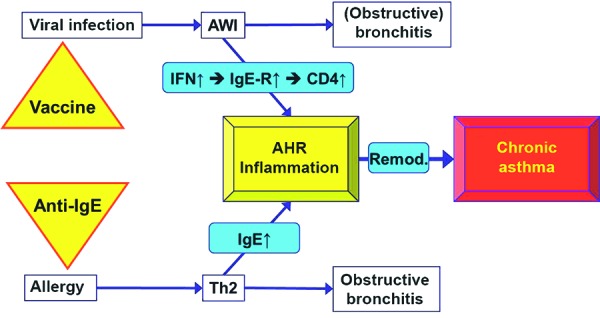
Dual mechanism of the development of persistent asthma. Viral infections and allergic sensitization potentiate each other’s effect on the induction of Th2-directed allergic airway inflammation and offer approaches to develop innovative, preventive, and therapeutic options.

Thus, a model as shown in Figure 3 can be postulated: Allergic sensitization will induce the Th2 response as well as IgE-mediated immediate-type allergic reactions in the pulmonary tissue. Early viral infections of the lower airways lead to a further intensification of the allergic immune reaction and in particular to an expression of the IgE receptor, which further heats up the Th2 cascade (“Il-4/IL-13 storm”). Exacerbation of the existing inflammation based on allergic sensitization due to viral infections leads to early remodeling, which, in the end, is the pathological correlate of persistent airway inflammation and chronic bronchial asthma. 

## What can be learnt from the model of dual development of persistent airway inflammation? 

For pediatric allergists, the main task will be an early prevention of asthma and allergy [6]. With regard to the prevention of persistent asthma, there are two points of action (Figure 3):

- prevention of early viral infections of the lower airways,
- prevention of early allergic sensitization.

Currently, several clinical studies investigating an active vaccination against RSV are about to start. For HRV, the situation is significantly more difficult because there are numerous serotypes for which no common vaccination has been found. Nevertheless, a successful, active vaccination against RSV and HRV would significantly reduce the risk of developing persistent bronchial asthma! 

In addition, all measures against early allergic sensitization are certainly helpful in reducing the risk of developing persistent asthma. In this context, first clinical trials from the last years could demonstrate that unspecific immunomodulation, either by probiotics or by bacterial lysates [13, 18], can reduce the risk of allergic sensitization or the development of an allergic disease. These studies cannot yet be extrapolated to the general population, but they show that unspecific immunomodulation can indeed influence the early development of allergic sensitization.
